# Combination of Methoprene and Controlled Aeration to Manage Insects in Stored Wheat

**DOI:** 10.3390/insects7020025

**Published:** 2016-06-17

**Authors:** Samuel S. Liu, Frank H. Arthur, Douglas VanGundy, Thomas W. Phillips

**Affiliations:** 1 Department of Entomology and Plant Pathology, Oklahoma State University, Stillwater, OK 74078, USA; lswqy@yahoo.com; 2 Food Forensics Deptartment, Certified Laboratories, 200 Express St., Plainview, NY 11803, USA; 3 USDA, Agricultural Research Service, Center for Grain and Animal Health Research, 1515 College Avenue, Manhattan, KS 66502, USA; Frank.Arthur@ARS.USDA.GOV; 4 Central Life Sciences, 12111 Ford Rd., Dallas, TX 75234, USA; DVangundy@central.com; 5 Department of Entomology, Kansas State University, 123 W. Waters Hall, Manhattan, KS 66506, USA

**Keywords:** *Plodia interpunctella*, *Tribolium castaneum*, *Cryptolestes ferrugineus*, *Rhyzopertha dominica*, *Sitophilus oryzae*, stored products, insect growth regulator

## Abstract

A commercial formulation of the insect growth regulator methoprene was applied to wheat stored in small bins either alone or in combination with controlled aeration of the bins, to lower grain temperature for insect pest management of stored wheat. Grain temperatures were monitored and modified by a computer-controlled thermocouple system that also activated the aeration system at programmed set-points to move cool ambient air through the grain mass to lower grain temperature. Results from sampling insect populations in experimental storage bins along with laboratory mortality bioassays of insects placed on wheat taken from the bins over the course of the storage period showed that methoprene was very effective in controlling infestation by the externally-feeding stored grain insects *Plodia interpunctella* (Hübner), the Indian meal moth *Tribolium castaneum* (Herbst), the red flour beetle, *Cryptolestes ferrugineus* (Stephens), the rusty grain beetle, and also for the internal-feeding pest *Rhyzopertha dominica*( Fauvel), the lesser grain borer. Methoprene did not give good control of the internal-feeding pest *Sitophilus oryzae* (L.), the rice weevil. Aeration alone was somewhat effective in suppressing insect population development, while methoprene alone or when combined with aeration greatly enhanced insect control. Commercial grain grading for industry quality standards at the end of the storage period confirmed the impact of insect suppression on maintaining high quality of the stored wheat. This field experiment shows that methoprene combined with aeration to cool grain can be effective for pest management of stored wheat in the southern plains of the United States of America.

## 1. Introduction

The insect growth regulators (IGRs) are insecticides that mimic insect molting hormones and interfere with normal immature insect development [1,2,3]. IGRs include juvenile hormone analogues (JHAs) which are considered biopesticides because they generally have very low mammalian toxicity and can have limited impacts on non-target arthropod species [4]. IGRs interfere with insect metabolism in a manner that disrupts normal growth and development. Usually, the insect dies before it reaches full maturity. Insect growth regulators are typically selective for insects and can either inhibit the synthesis of chitin required for forming new cuticle at each molt, or disrupt or replace the production of juvenile hormones that regulate the molting process [5,6,7].

Methoprene is a synthetic JHA that interrupts insect growth and development and can control a number of stored product pests [1,8,9,10,11]. The formulation Diacon^®^ (Central Life Sciences, Schaumberg, IL, USA) was registered during the 1980s in the USA but it was not widely used [10]. A later commercial formulation, Diacon II^®^ (formulated at 288 mg/mL active ingredient, AI), which contains the s-isomer of methoprene, was registered as a grain protectant in the US in 2002 and can be applied at rates of 1.0, 2.5 or 5.0 ppm on various grains and nuts [3].

Aeration with ambient air is an important component of integrated pest management strategies to help manage insect infestation in stored grain by cooling the grain [12,13,14,15,16]. Aeration slows down the normal development of immature insects by lowering the ambient temperature. However, aeration cooling will rarely kill insects in stored grain as acutely lethal temperatures are not attained [16,17]. Most stored grain insect pest species do not reproduce at grain temperatures lower than 15 °C [18,19]. Although aeration does not cause acute mortality, it is effective in controlling stored product pest populations by reducing temperatures in the grain mass and is thus recommended to control stored grain insects [4,20].

Residual degradation of insecticides used as grain protectants, especially older organophosphates, increases with temperature [14]. Although methoprene is relatively stable at different temperatures the residual activity and efficacy could be even further enhanced in aeration-cooled grain compared to non-aerated grain when stored under hot ambient conditions in the field [21]. Various laboratory studies that evaluated efficacy of IGRs as aerosols, contact surface treatments, or layer treatments (e.g., top of grain compared to other applications) to control stored product insects and some recent studies have evaluated efficacy of methoprene under field conditions [3,21,22,23,24]. However, there are no scientific field studies that researched the efficacy of IGRs combined with aeration cooling of stored grain. The objectives of this current study were to: (1) evaluate efficacy of methoprene for insect pest control when applied as a top-layer or as complete bin treatment to stored wheat, either or without aeration cooling over 40 weeks of storage; (2) evaluate the impacts of methoprene-aeration treatments on grain quality over the storage period; and (3) determine the stability of methoprene residues under these same treatment and storage conditions.

## 2. Materials and Methods

### 2.1. Methoprene Application, Grain Storage and Quality Evaluations

The methoprene used in the field trials was the former commercial product Diacon II^®^ Emulsifiable Concentrate (EC) (288 mg AI/mL, Central Sciences International, Schaumburg, IL, USA). The concentrated insecticide was diluted in water according to manufacturer’s instructions and applied to the grain at 19.0 L/t, which was to result in a calculated dosage of 1.0 ppm by weight applied to grain. The sprayer used for the test was a “Little Gus” field sprayer, originally sold by Gustfason Inc., Plano, TX (now a part of Bayer Corporation, Pittsburg PA, USA). Sixteen corrugated round steel bins with a storage volume of approximately 17.6 m^3^, adequate to hold 13.6 MT of wheat, were used for the field trial, which was done at the Stored Product Research and Education Center of Oklahoma State University, Stillwater, OK, USA. All bins were equipped with a raised ventilated floor and an aeration fan in the “push” orientation, such that ambient outside air could be drawn into the bin, up through the floor and into the grain mass during a cooling cycle. The lids at the top openings of the aerated bins were propped up to an opening of about 3 cm at the side opposite the hinges to allow cooling air from aeration to escape. Grain temperatures were automatically monitored and recorded hourly and the aeration fans were turned on for the aeration-treated bins using an aeration control system from OPI Systems Inc. (Calgary, Alberta, Canada). Fans for the designated aeration bins were set to activate when the outside air temperature at 3 m above ground was 5 °C lower than the grain temperature at 30 cm below the grain surface. Grain for this experiment was newly harvested hard red winter wheat, *Triticum aestvium*, purchased from a local producer and delivered directly from the field at the time of harvest with no application of any chemical before loading into our bins on 21 July. The following four treatments were randomly assigned to each of four bins: (1) aeration only with no methoprene as our experimental control; (2) methoprene at 1.0 ppm applied to the entire grain mass with no aeration; (3) methoprene at 1.0 ppm applied just to the top 50 cm of the grain mass, with no aeration; and (4) a combination of 1.0 ppm methoprene applied to the entire grain mass plus controlled aeration. A truly untreated experimental control, for which there would be no aeration and no application of methoprene, was not used for this experiment due to concerns with over-infestation of valuable wheat in such a true control, our preference for adequate and balanced replication (n = 4), and our desire to compare efficacy of methoprene under cool grain (aerated) and warm grain (non-aerated) conditions.

The grain flow rate while loading into bins was calibrated (kg/min) when the aeration-only bins were loaded, and this value was used to calculate appropriate application of the methoprene solution to reach the target application rate of 1 ppm. Individual volumes of solution were prepared for each bin involving methoprene. Methoprene spray treatments were applied directly to grain as it was loaded into bins by spraying the diluted product onto grain as it was dumped from a truck into the collection hopper at the bottom of a 15-cm screw auger conveyor that elevated the grain into the bins at the top-center capped opening on the roof.

Official grain grading and methoprene residue analyses were carried out at both the beginning and the end of this study. Samples (each >1.0 kg) from each bin were sent to a commercial service laboratory (Enid Grain Inspection Company, Enid, OK, USA) for grading, and to Central Life Sciences to analyze for methoprene residues via established methods [25].

### 2.2. Insect Introductions to Bins

*Tribolium castaneum* (Herbst) (Coleoptera: Tenebrionidae), the red flour beetle, (*Rhyzopertha dominica* (Coleoptera: Bostrichidae), the lesser grain borer, and *Cryptolestes ferrugineus* (Stephens) (Coleoptera: Laemophloeidae), the rusty grain beetle, were added to bins at various times after the bins were loaded with the wheat. The insects were obtained from stock pesticide-susceptible laboratory cultures maintained at Oklahoma State University, (OSU) Stillwater, OK, USA. *T. castaneum* was reared on 400 cm^3^ of whole hard red winter wheat flour plus 2.5 cm^3^ yeast in 0.95 L glass containers. *R. dominica* was reared on 400 g sound wheat containing 4% yeast in each container, while *C. ferrugineus* was reared on 400 cm^3^ rolled oats with 5 cm^3^ whole hard red winter wheat flour and 2.5 cm^3^ yeast in each container. All species were maintained in growth chambers at 28–30 °C and 65 ± 5% r.h. One hundred mixed-sex adults of each of these three species were added to each of the 12 bins at 7, 14, 21 and 28 days after the wheat was loaded into the bins. Insect samples from probe traps and also insect counts from direct grain samples were made at day 28. One WB-II probe trap (Trece Inc., Adair, Oklahoma, USA) was inserted into the top 30 cm of grain in each bin and kept there for seven days. Captured adult insects were then identified to species and counted. Grain samples of 1.0–2.0 kg were collected from each bin using a 1.5-m-long brass open-handle probe, referred to as a “grain trier” (Seedburo Equipment Co., Des Plaines, IL, USA). Grain trier samples were taken from the top of the grain in each bin and then weighed and sifted for identification and counting of adult insects. Trap and grain samples were collected from all 16 bins at 4, 8, 12, 16, 20 and 40 weeks after the bins were loaded at the start of the study.

### 2.3. Laboratory Bioassays

*Plodia interpunctella* (Hübner) (Lepidoptera: Pyralidae), the Indian Meal moth, *T. castaneum*, *R. dominica*, and *Sitophilus oryzae*, (L.) (Coleoptera: Curculionidae) the rice weevil were used for the laboratory bioassay of treated grain from bins. The colonies of these four insect species were reared in growth chambers. *P. interpunctella* was reared in 0.95 Liter glass jars on a diet of 37% cornmeal, 25% egg crumbles, 25% chick starter, and 13% glycerin (v:v) at 28 °C, 65 ± 5% r.h., and a 16h/8h light/dark cycle. *T. castaneum* and *R. dominica* was reared as described for the field study. *Sitophilus oryzae* was reared on 400 cm^3^ sound wheat plus 2.5 cm^3^ of yeast in the same containers and rearing conditions as previously described for the other species reared on wheat. 

Grain used for laboratory bioassays was taken directly from the field study grain bins at three different times after treatment: one day after loading (21 July) and then after 20 (9 December) weeks and 40 weeks (28 April) of storage. *P. interpunctella* eggs that were 0–24-h-old were collected from rearing jars containing 1–3-d-old moths. Twenty *P. interpunctella* eggs attached to double-sided sticky tape on black paper were placed in a each of 120-mL jars containing 20 g of sound wheat kernels plus 20 g crushed wheat from each bin, and maintained at 28 °C and 65 ± 5% r.h. with a 16 h/8 h light/dark photoperiod in a growth chamber. Egg hatch was checked after 1 week, and counts of normal adults were made after 6, 7 and 8 week. Fifty *T. castaneum*, *R. dominica*, and *S. oryzae* adults were separately placed into 240-mL jars each containing 100 g wheat (5 g crushed for *T. castaneum*) from each bin and maintained at 28 °C and 65 ± 5% r.h. under a 16h/8h light/dark photoperiod in a growth chamber. After 1 week the parent adults were removed from each jar and placed in individually clean Petri dishes for a recovery period of 24 h, and then counted to determine mortality. After 6 weeks the F1 progeny from the grain were sifted and counted.

### 2.4. Statistical Analysis

Data sets were analyzed separately for each species or treatment using the General Linear Models procedure (SAS Institute, Cary, NC, USA). All data were subjected to analysis of variance (ANOVA) followed by a protected least significant difference (LSD) test for comparison of means. Data were transformed by square root of the number plus 0.5 prior to the ANOVA analysis, while non-transformed means and standard errors (SE) are reported below. Significant biological differences were considered at the *p* < 0.05 level.

## 3. Results

### 3.1. Treatment Effects on the Stored Wheat

**Figure 1:**
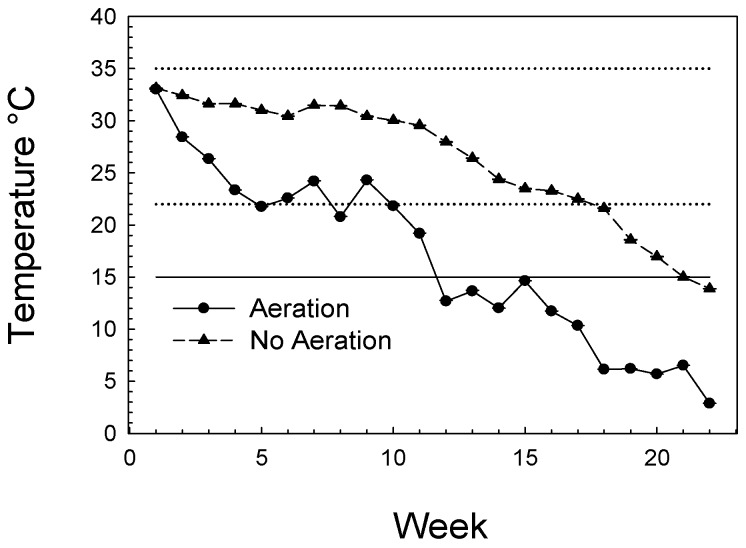
Mean temperature (°C) each week recorded at one m below the surface in aerated and non-aerated bins (N = 8 bins of each) for 22 weeks of the study; standard errors of the weekly means were not greater than 2 °C in any case and are not shown in the figure. Aeration began on 21 July and Week 1 mean temperatures were recorded on 1 August. Data are shown to week 22, which ended 16 January. Solid horizontal line at 15 °C is the minimum temperature for development of the species studies; dashed lines indicate their optimum development range of 22 °C to 35 °C.

Controlled aeration applied to the small bins of wheat for two of the treatments, eight bins total, caused a distinct cooling of grain when compared to grain temperatures for the eight bins without any aeration (Figure 1). Grain in the aerated bins averaged about 6 degrees lower than grain in unaerated bins after one week of cooling, and achieved about a 10 degree difference that was maintained for the course of the study. At the end of the 22nd week, on 16 December, the temperatures in aerated bins was about 3 °C while temperatures in unaerated bins were about 14 °C.

Wheat samples from the 12 bins treated with methoprene had residues that varied from about 0.53 ppm in the top-treated bins to a range of 1.0 to 2.0 ppm in completely treated bins that were measured at the start of the study just after bin-loading. The average proportion (±SE of the means) of methoprene residue remaining at the end of the 40-week study was about 36.9% (±3.8) for the top-treated bins, 71.4% (±8.9) for the methoprene only bins, and 68.7% (±10.7) for the bins with methoprene plus aeration. Although grain temperatures were warmer in non-aerated bins, which could have caused some degradation of the applied methoprene, there were no significant differences in methoprene residues between aerated in non-aerated bins (ANOVA, *p* > 0.05).

Grading results showed that the bulk density of the wheat and the official quality grades had not decreased after 10 months of storage for any of the treatments applied to these bins. All the sampled what was determined to have a U.S. grade = 1 for all bins at the end of the study. If we had included a control set of bins with no aeration and no methoprene we suspect that those bins would have suffered a reduced quality score to insect-associated factors. However, as explained above, we were unable to include such a control in this experiment. The average numbers of insect-damaged kernels (±SE of the means) per 100 gram (IDK) was 9.0 (±4.2) in the aeration only treatment, 4.3 (±1.3) and 3.3 (±1.3) mean IDK in the top-layer methoprene and methoprene alone treatments, respectively; but there were only 2.0 IDK (±1.0) on average in the methoprene combined with aeration treatment.

### 3.2. Insect Populations in Bins

Table 1, Table 2, Table 3, Table 4, Table 5 and Table 6 report the mean numbers of insects in samples taken from the bins at various time intervals during the 40-week study. Probe traps samples (Table 1, Table 3 and Table 5) report numbers trapped during one weeks in the top of each bin, whole grain trier samples are the numbers of insects in an approximately 1-kg sample of grain taken from each bin. Insect numbers overall were higher in samples taken during the warmer times of week 4, week 8 and week 12, after which the numbers were lower as temperatures reduced insect activity. In cases for which there were statistically significant differences among the treatments the highest insect counts were in the bins treated with aeration only.

There were significant differences among treatments for numbers of *T. castaneum* adults in probe traps at eight weeks, 12 weeks and 40 weeks (ANOVA, *p* < 0.05) with the lowest numbers captured in bins completely treated with methoprene and under aeration (Table 1). Adults trapped in the aeration-only bins averaged 1,017 and 1,975 at 4 weeks (August) and 8 weeks (September), and these numbers are tremendously higher than expected given that only 400 adults had been added to each bin during the first four weeks of storage. Thus, high numbers of *T. castaneum* likely resulted from field immigrants to these bins. Nevertheless, by week 12 the population suppression effect of the total methoprene treatment as a growth regulator was seen in comparison to the methoprene top and aeration only treatments. *T. castaneum* adults collected in grain trier samples of 1 kg of wheat showed that there were significant differences in mean adult numbers among the different treatments taken at the first three sampling periods, with the lowest beetle densities being in treatments that had methoprene applied to the entire grain mass (Table 2). of 4, 8 and 12 weeks but there were no significant changes in mean adult numbers among all treatments taken at the last three sampling months (*p* > 0.2) (Table 2).

Numbers of *R. dominica* adults caught in probe traps showed that there were no significant treatment effects (*p* > 0.05) in the first five sampling dates, but the last sample time of 20 weeks, at the end of the study, showed significantly more beetles in the aeration plus methoprene bins (Table 3). The trap captures for *R. dominica* were very low compared to those for *T. castaneum*, which probably reflects the differences in mobility between adults of these two species. Insects counted directly from grain trier samples gave a direct measurement of insect density at the time of sampling (Table 4), and in five out the six sample times there were significant treatment effects with the lowest insect numbers found in the methoprene only and methoprene plus aeration bins.

The mean numbers of *C. ferrugineus* adults recovered by probe traps and in grain trier samples are shown in Table 5 and Table 6, respectively. There were no significant differences among treatments for mean adult numbers in probe traps for the first three sampling periods, but there were significant treatment effects seen in the last three periods with total methoprene treatments having the lowest numbers of *C. ferrugineus*. Nevertheless, there were significant differences in mean adult numbers for all treatments at the last three sampling months (Table 5). Grain trier samples of grain showed treatment effects for numbers of *C. ferrugineus* at each of the six sampling times (Table 6), with the highest numbers in the aeration only bins and substantially lower numbers in bins that had any of the methoprene treatments.

### 3.3. Laboratory Bioassays of Treated Grain from Bins

There was no mortality of any of the parent adult beetles added to experimental grain that had been collected just after bin-loading, 20 weeks and 40 weeks after loading, whether on untreated wheat (aeration only) or methoprene-treated wheat, following the one week of exposure during controlled laboratory infestations. Adult progeny emergence from these samples exposed to laboratory infestations of beetles are reported in Table 7. There was just one T *castaneum* adult produced in one jar of wheat taken from an aeration + methoprene bin 40-weeks after treatment, while there were no adults produced in any of the other samples that had been treated with methoprene. Results were similar for *R. dominica* exposed to wheat taken from bins one day after loading, for which there were only a few beetles produced in a few jars. At 20 weeks and 40 weeks there were low number of *R. dominica* produced in the grain that had the methoprene top only treatment, while nearly zero *R. dominica* were generated in wheat from bins treated entirely with methoprene. Bioassays using *S. oryzae* were markedly different than those with the other two beetle species as hundreds of beetles were generated from all samples of grain in this experiment. Significant treatment effects (*p* < 0.01) for *S. oryzae* progeny production were found only for the 20-week old grain, although all means were quite high, ranging from 430.5 to 681.3.

Our bioassays for effects of treated grain on the development of *P. interpunctella* following inoculation of jars with 20 eggs showed that adult emergence was nearly 100%, with 19 or 20 emerging per jar, for wheat taken form the aeration-only bins at one day, 20 weeks or 40 weeks of storage (Table 8). Wheat from bins receiving the top-only treatment of methoprene had only 3.9% emergence from grain sampled at 1-day after storage and then a slight increase to approximately 8% and 22% at the longer storage periods of 20 weeks and 40 weeks, respectively. Wheat from bins treated entirely with methoprene had almost no adult emergence, only two adults at 40 weeks, from the grain sampled at any of the three time periods.

## 4. Discussion

The research reported here may represent the first controlled field trial of methoprene applied to bulk-stored grain over a long storage period. The work demonstrates the residual activity of methoprene as an IGR during grain storage and the results suggest that methoprene can reduce pest population increases and associated grain damage over time. Data collected on grain temperatures clearly show that controlled aeration can reduce the temperature of stored wheat beginning in just days from the start of storage and then achieve a 10-dgree difference between aerated and non-aerated bins for over 20 weeks. Lower grain temperatures like those achieved here can double or triple the egg-to-adult development times of grain pests compared to those of warmer unaerated grain, resulting in substantial decreases in the rate of pest population growth [4]. Insect populations measured with probe traps and direct grain samples were generally the highest in our aerated bins, but we did not have a non-methoprene with no-aeration as a true control for comparison. One objective for studying the combination of aeration cooling with methoprene application was to see if pest population growth and could be lower and IGR activity higher from grain in those combination bins compared to methoprene-only bins. However, numbers of the three beetle species studied did not differ between these two bin treatments in either probe trap samples or direct grain sample at any of the sampling periods. Similarly, IGR activity tested in laboratory assays against four species showed no improved activity from aeration. Our insect data therefore suggest that levels of methoprene on aerated and non-aerated wheat were similar. This conclusion is validated by our chemical residue analyses that found the percentage of methoprene remaining on non-aerated wheat was not statistically different from that on aerated wheat at the end of the 40-week study.

Insect numbers in probe traps and those collected from direct grain samples provided information on variation of insect populations over time and among the bins with different experimental treatments related to methoprene and aeration. Grain probe traps are relative sampling tools that are biased by mobility of the insects being trapped, compared to grain samples that give a one-time measure of insects present in that grain sample at the time of collection. Insects with mobile adults that move and disperse actively through a grain mass, such as *T. castaneum* and *C. ferrugineus* studied here, will be caught at higher numbers in probe traps compared to a less mobile species like *R. dominica* [26]. Our experiment found that numbers of T *castaneum* and *C. ferrugineus* from both probe traps and grain samples were significantly reduced in most bins with methoprene during one or more sampling periods of this study (Table 1, Table 2, Table 5 and Table 6). The numbers of *R. dominica* adults however, were quite low in both probe trap samples and grain samples, but it was the grain samples that showed significant methoprene effects during several weeks (Table 3 and Table 4) Interestingly, the numbers of T *castaneum* in probe traps were much higher than expected at the first sampling time, four weeks after bin-filling. Each of our 16 bins had 100 adults of the three beetle species added weekly, for a total of 400 adults added by week 4, but probe trap number in the aerated-only bins averaged over 1000 beetles, suggesting than several thousand adult *T. castaneum* were in each bin at week 4. These high numbers were unlikely to have resulted from reproduction in that 4-week period; the warm grain temperatures of 30–32 °C in those non-methoprene bins during that time likely did not contribute to generating 1000’s of new adults based on development rates of those species [13]. Bins were clean and free of grain before loading, so residual *T. castaneum* are not implicated in contributing to these high numbers. More likely is the possibility that feral immigrant *T. castaneum* adults entered the bins via openings used in aeration and ventilation (e.g., that partially opened top loading port), and perhaps stimulated by the naturally produced aggregation pheromones emitted by the resident beetles [27].

Our laboratory bioassays demonstrated that activity of methoprene as an IGR was retained for up to 40 weeks in bins of wheat treated at 1 ppm just on the top 50 cm or with the entire grain mass treated at 1 ppm (Table 7 and Table 8). All grain samples used for the laboratory assays were collected with a grain sampler to a depth of 1.5 m, so it is understandable that the samples from the top-treated methoprene bins had less residual methoprene than those from completed treated bins. Bioassays with *P.* interpuntella, *T. castaneum* and *R. dominica* had low or completely no progeny produced in wheat from bins with either top-treated or completely treated methoprene application, and top-treated wheat had intermediate but very low numbers of adult progeny for *P.* interpuctella and *R. dominica*. The partial effectiveness against *R. dominica* in bioassays done at 20 and 40 weeks is likely due to females of this species laying eggs in the grain mass outside of kernels, where neonate larvae may receive an effective dose of methoprene, followed by good survival from unaffected larvae that bore into a kernel to complete development where they escape the methoprene. Other studies also reported methoprene control of *R. dominica* on wheat and on rice [9,10]. Implications for sub-optimal applications of a “top-dressing” to grain with a residual insecticide were also suggested in laboratory studies that used the desiccant diatomaceous earth, DE [28]. In that study, *R. dominica* were able to penetrate through a top layer of wheat treated with diatomaceous earth (DE) and oviposit in the untreated wheat below the treated layer, even though some adult females later died from the exposure to DE. Progeny of *S. oryzae* were plentiful after exposure of treated wheat to 50 adults for one week in our bioassays, and showed no expected treatment-related effects in these laboratory bioassays. These results with *S. oryzae* can be explained by the fact that methoprene is not acutely toxic to adult insects and this species deposits its eggs and completes full develop inside a kernel of wheat where no methoprene is present to impair development. This situation that renders methoprene ineffective against *Sitophilus oryzae* was also demonstrated by [29].

The work reported here contributes to the growing number of studies on IGRs for stored product insect pests. Wheat in the USA is usually stored for 12-36 weeks in farm bins [30] and our study clearly shows that methoprene applied to wheat at 1.0 ppm can provide protection from insect infestation for at least 40 weeks based on the insect numbers resulting in the infested bins, effective IGR activity for 40weeks in the treated grain, methoprene residue analyses and lower grain damage of the treated wheat. Methoprene does not, however, control all grain insect pests, so alternative or additional pest control methods may be needed [24], and its use for maanging resistance to otther insecticides should be approached with caution. Methoprene is a synthetic mimic of insect juvenile hormone and therefore could be of interest for resistance management since it likely has a mode of action very different from most chemical insecticides in use. However, resistance to methoprene was documented in *Lasioderma serricorne* (F.) (Coleoptera: Anobiidae) as early as 1994 [31], and methoprene resistance in other species of stored grain insects was reported in recent years (e.g., [32]). Nevertheless, as with any insecticide, judicious use of methoprene in stored grain integrated pest management can be effective and could serve tocombat serious pests while also reducing overall chemical inputs [4,11].

## 5. Conclusions

This research clearly shows that application of methoprene to stored wheat at the time of bin-loading can protect that grain from insect infestation throughout 40 weeks of storage. The experiment confirms that thermostatically-controlled aeration to stored wheat, such that aeration fans turn on only when outside temperatures are cooler than those within the grain mass, can result in substantial grain cooling that can deter development of infesting insects. The high level of insecticidal activity of the methoprene-treated wheat in our experiments, whether from aerated or non-aerated bins, was essentially unchanged throughout 40 weeks of storage in these field bins. Methoprene applied to just the top 50 cm of the grain elicited significant insect control compared to untreated wheat, but admixture of methoprene to the entire grain mass had the best results overall. This research provides one of the few controlled field validations of previous laboratory studies that suggest the application of methoprene onto commercially-stored grain can be an effective alternative to the use of other traditional insecticides and fumigants.

## Figures and Tables

**Table 1:** Mean numbers (±SE) of adult *T. castaneum* captured by probe traps at different times after bins were loaded with wheat that was either treated with aeration only, the top 50 cm-treated with methoprene, methoprene treatment to all the wheat in a bin, or aeration plus complete treatment with methoprene on all grain in the bin. Means within columns with the same upper case letter, for which there was a significant (*p* ≤ 0.05) F-statistic, are not significantly different (least significant difference (LSD) test, *p* < 0.05, SAS Institute, degrees of freedom for analysis within date for treatment were 3, 12).

Treatments	4 Weeks	8 Weeks	12 Weeks	16 Weeks	20 Weeks	40 Weeks
Aer. Only	1017.5 ± 227.6	1974.7 ± 491.7 ^A^	41.7 ± 16.9 ^A^	22.0 ± 11.7	79.0 ± 50.5	2.5 ± 1.0 ^A^
Meth. Top	660.1 ± 242.5	974.2 ± 473.7 ^AB^	153.0 ± 79.3 ^A^	46.0 ± 28.1	33.7 ± 30.4	1.5 ± 0.9 ^B^
Meth. Total	772.5 ± 383.4	340.2 ± 340.2 ^B^	24.2 ± 7.5 ^B^	5.0 ± 1.0	1.7 ± 1.4	0.2 ± 0.2 ^B^
Aer. + Meth.	250.2 ± 33.2	301.0 ± 73.3 ^B^	7.2 ± 10.1 ^B^	5.5 ± 1.3	0.0 ± 0.0	0.0 ± 0.0 ^B^
*F* and *p*	2.1, 0.16	5.1, 0.02	4.2, 0.03	2.4, 0.12	1.6, 0.25	3.3, 0.05

**Table 2:** Mean numbers (±SE) of adult T. castaneum collected in grain trier samples at different times after bins were loaded with wheat that was either treated with aeration only, the top 50 cm-treated with methoprene, methoprene treatment to all the wheat in a bin, or aeration plus complete treatment with methoprene on all grain in the bin. Means within columns with the same upper case letter, for which there was a significant (*p* < 0.05) F-statistic, are not significantly different (LSD test, *p* < 0.05, SAS Institute, degrees of freedom for analysis within date for treatment were 3, 12).

Treatments	4 Weeks	8 Weeks	12 Weeks	16 Weeks	20 Weeks	40 Weeks
**Aer. Only**	11.7 ± 227.6 ^A^	5.7 ± 2.2 ^A^	5.1 ± 1.8 ^A^	6.4 ± 4.7	3.4 ± 2.2	2.5 ± 1.0
**Meth. Top**	0.8 ± 0.4 ^B^	2.2 ± 0.7 ^A^	3.0 ± 1.1 ^AB^	1.5 ± 1.5	1.5 ± 0.8	0.0 ± 0.0
**Meth. Total**	1.05 ± 0.5 ^B^	0.8 ± 0.6 ^B^	1.0 ± 0.4 ^BC^	0.6 ± 0.6	0.2 ± 0.2	0.0 ± 0.0
**Aer. + Meth.**	0.6 ± 0.4 ^B^	1.9 ± 0.5 ^B^	0.4 ± 0.4 ^C^	0.6 ± 0.4	0.6 ± 0.3	0.0 ± 0.0
***F* and *p***	23.5, <0.01	4.3, 0.03	4.7, 0.02	1.4, 0.28	1.6, 0.25	1.0, 0.42

**Table 3:** Mean numbers (±SE) of adult R.dominica captured by probe traps at different times after bins were loaded with wheat that was either treated with aeration only, the top 50 cm-treated with methoprene, methoprene treatment to all the wheat in a bin, or aeration plus complete treatment with methoprene on all grain in the bin. Means within columns with the same upper case letter, for which there was a significant (*p* < 0.05) F-statistic, are not significantly different(LSD test, *p* < 0.05, SAS Institute, degrees of freedom for analysis within date for treatment were 3, 12).

Treatments	4 Weeks	8 Weeks	12 Weeks	16 Weeks	20 Weeks	40 Weeks
**Aer. Only**	1.2 ± 1.0	1.7 ± 0.6	3.5 ± 1.0	1.2 ± 0.8	9.7 ± 8.7	0.2 ± 0.2 ^B^
**Meth. Top**	1.2 ± 1.0	1.5 ± 1.0	2.7 ± 1.8	0.0 ± 0.0	17.8 ± 9.8	0.0 ± 0.0 ^B^
**Meth. Total**	3.7 ± 0.5	0.2 ± 0.2	0.5 ± 0.5	0.5 ± 0.5	1.2 ± 0.5	0.8 ±0.5 ^AB^
**Aer. + Meth.**	4.5 ± 1.8	0.2 ± 0.2	0.5 ± 0.3	0.5 ± 0.5	2.2 ± 0.7	2.0 ± 0.7 ^A^
***F* and *p***	0.9, 0.46	1.6, 0.23	2.7, 0.10	1.14, 0.40	2.1, 0.16	4.53, 0.02

**Table 4:** Mean numbers (±SE) of adult R. dominica collected in grain trier samples at different times after bins were loaded with wheat that was either treated with aeration only, the top 50 cm-treated with methoprene, methoprene treatment to all the wheat in a bin, or aeration plus complete treatment with methoprene on all grain in the bin. Means within columns with the same upper case letter, for which there was a significant (*p* < 0.05) F-statistic, are not significantly different (LSD test, *p* < 0.05, SAS Institute, degrees of freedom for analysis within date for treatment were 3, 12).

Treatments	4 Weeks	8 Weeks	12 Weeks	16 Weeks	20 Weeks	40 Weeks
**Aer. Only**	2.7 ± 0.7	2.8 ± 0.9 ^AB^	4.4 ± 1.1 ^A^	2.4 ± 1.0	3.8 ± 1.6 ^A^	2.5 ± 1.0
**Meth. Top**	0.6 ± 0.2	4.0 ± 1.4 ^A^	2.2 ± 1.5 ^AB^	2.0 ± 1.1	1.3 ± 0.6 ^B^	0.0 ± 0.0
**Meth. Total**	1.3 ± 0.4	1.0 ± 0.7 ^B^	0.8 ± 0.4 ^B^	0.2 ± 0.2	0.6 ± 0.4 ^BC^	0.0 ± 0.0
**Aer. + Meth.**	1.7 ± 0.5	0.9 ± 0.5 ^B^	1.1 ± 0.5 ^AB^	0.4 ± 0.2	0.0 ± 0.0 ^C^	0.0 ± 0.0
***F* and *p***	2.2, 0.14	2.9, 0.08	2.9, 0.08	2.47, 0.11	15.6, <0.01	0.8, 0.50

**Table 5:** Mean numbers (±SE) of adult C. ferrugineus captured by probe traps at different times after bins were loaded with wheat that was either treated with aeration only, the top 50 cm-treated with methoprene, methoprene treatment to all the wheat in a bin, or aeration plus complete treatment with methoprene on all grain in the bin. Means within columns with the same upper case letter, for which there was a significant (*p* < 0.05) F-statistic, are not significantly different (LSD test, *p* < 0.05, SAS Institute, degrees of freedom for analysis within date for treatment were 3, 12).

Treatments	4 Weeks	8 Weeks	12 Weeks	16 Weeks	20 Weeks	40 Weeks
**Aer. Only**	40.0 ± 13.4	34.0 ± 0.6	26.20 ± 9.6	24.2 ± 13.4 ^A^	5.2 ± 1.9 ^A^	43.5 ± 12.32 ^A^
**Meth. Top**	29.0 ± 10.8	20.0 ± 1.0	14.50 ± 11.2	7.0 ± 1.7 ^A^	7.2 ± 2.5 ^A^	11.5 ± 10.2 ^B^
**Meth. Total**	45.8 ± 8.5	28.7 ± 0.2	2.8 ± 1.0	1.5 ± 0.9 ^B^	0.0 ± 0.0 ^B^	0.0 ± 0.0 ^B^
**Aer. + Meth.**	43.5 ± 28.2	25.3 ± 7.0	5.2 ± 2.6	3.2 ± 2.1 ^B^	0.8 ± 0.8 ^B^	1.0 ± 0.4 ^B^
***F* and *p***	0.2, 0.87	0.2, 0.90	2.4, 0.12	4.1, 0.03	6.8, <0.01	4.53, 0.02

**Table 6:** Mean numbers (±SE) of adult C. ferrugineus collected in grain trier samples at different times after bins were loaded with wheat that was either treated with aeration only, the top 50 cm-treated with methoprene, methoprene treatment to all the wheat in a bin, or aeration plus complete treatment with methoprene on all grain in the bin. Means within columns with the same upper case letter, for which there was a significant (*p* < 0.05) F-statistic, are not significantly different (LSD test, *p* < 0.05, SAS Institute, degrees of freedom for analysis within date for treatment were 3, 12).

Treatments	4 Weeks	8 Weeks	12 Weeks	16 Weeks	20 Weeks	40 Weeks
Aer. Only	6.4 ± 2.8 ^A^	17.7 ± 2.2 ^A^	12.0 ± 3.5 ^A^	22.4 ± 12.3 ^A^	31.0 ± 3.8 ^A^	7.0 ± 2.3 ^A^
Meth. Top	1.7 ± 0.9 ^AB^	1.5 ± 1.0 ^B^	0.6 ± 0.4 ^B^	2.9 ± 1.8 ^AB^	3.1 ± 1.2 ^B^	0.0 ± 0.0 ^B^
Meth. Total	1.0 ± 0.2 ^AB^	0.4 ± 0.4b ^B^	0.0 ± 0.0 ^B^	0.0 ± 0.0 ^B^	0.0 ± 0.0 ^C^	0.0 ± 0.0 ^B^
Aer. + Meth.	1.0 ± 0.6 ^B^	1.5± 0.8 ^B^	0.2 ± 0.2 ^B^	1.5 ± 0.7 ^B^	0.0 ± 0.0 ^C^	0.0 ± 0.0 ^B^
*F* and *p*	2.6, 0.10	33.69, < 0.01	23.0, < 0.01	2.6, 0.06	6.8, <0.01	27.2, <0.01

**Table 7:** Mean numbers (±SE) of F1 progeny from 50 parental adults of either *T. castaneum*, *R. dominica*, and *S. oryzae exposed* on 100 g of wheat one day after treatment in bins, 20 weeks and 40 weeks after treatment. Means for each species within a column having the same upper case letter, for which there was a significant (*p* < 0.05) F-statistic, are not significantly different (LSD test, *p* < 0.05, SAS Institute, degrees of freedom for analysis within date for treatment were 3, 12).

Species	Treatment	1 Day	40 Weeks	40 Weeks
*T. castaneum*	Aeration Only	248.5 ± 14.3 ^A^	133.2 ± 22.7 ^A^	278.5 ± 2.2 ^A^
	Methoprene Top Only	0.0 ± 0.0 ^B^	0.0 ± 0.0 ^B^	0.0 ± 0.0 ^B^
	Methoprene Total	0.0 ± 0.0 ^B^	0.0 ± 0.0 ^B^	0.0 ± 0.0 ^B^
	Aeration + Methoprene	0.0 ± 0.0 ^B^	0.0 ± 0.0 ^B^	0.3 ± 0.2 ^B^
	F and P values	1096, < 0.01	105, < 0.01	352, < 0.01
*R. dominica*	Aeration Only	696.7 ± 37.9 ^A^	482.0 ± 22.7 ^B^	514.7 ± 2.2 ^A^
	Methoprene Top Only	1.5 ± 1.2 ^B^	31.2 ± 25.6 ^B^	25.2 ± 8.4 ^B^
	Methoprene Total	0.0 ± 0.0 ^B^	0.7 ± 0.7 ^B^	0.2 ± 0.2 ^C^
	Aeration + Methoprene	0.5 ± 0.5 ^B^	0.2 ± 0.2 ^B^	0.2 ± 0.2 ^C^
	F and P values	926, < 0.01	105, < 0.01	352, < 0.01
*S. oryzae*	Aeration Only	680.5 ± 37.9	598.8 ± 22.7 ^AB^	724.2 ± 55.9
	Methoprene Top Only	825.7 ± 207.2	430.5 ± 53.9 ^B^	517.7 ± 81.6
	Methoprene Total	1260.0 ± 588.6	582.3 ± 34.3 ^AB^	487.0 ± 50.8
	Aeration + Methoprene	464.5 ± 133.5	681.3 ± 61.2 ^A^	680.0 ± 100.2
	*F* and *p* values	1.0, 0.42	5.4, <0.01	2.4, 0.12

**Table 8:** Mean percentage (±SE) of *P. interpunctella* adults emerging (from addition of 20 eggs per jar) from 40 g-samples of wheat taken from bins at one day after treatment, 20 weeks and 40 weeks post-treatment. Means within a column with same letter, for which there was a significant (*p* < 0.05) F-statistic, are not significantly different (LSD test, *p* < 0.05, SAS Institute, degrees of freedom for analysis within date for treatment were 3, 12).

Treatment	1 Day	20 Weeks	40 Weeks
Aeration Only	98.7 ± 1.3 ^A^	98.8 ± 1.3 ^A^	98.8 ± 1.3 ^A^
Methoprene Top Only	3.9± 2.5 ^B^	8.0 ± 5.1 ^B^	21.8±10.6 ^B^
Methoprene Total	0.0 ± 0.0 ^C^	0.0 ± 0.0 ^C^	0.0 ± 0.0 ^C^
Aeration + Methoprene	0.0 ± 0.0 ^C^	0.0 ± 0.0 ^C^	0.0 ± 0.0 ^C^
*F* and *p* values	190.5, <0.01	79.4, <0.01	41.1, <0.01
